# Protein arginine methyltransferase 1 stimulates basal cell proliferation and migration to maintain corneal epithelial homeostasis

**DOI:** 10.1038/s41420-025-02684-6

**Published:** 2025-08-15

**Authors:** Jia Yang, Mingzheng Hu, Mulin Yang, Hua Ni, Jun Zhou, Dengwen Li, Jie Ran, Min Liu

**Affiliations:** 1 Department of Genetics and Cell Biology, College of Life Sciences, State Key Laboratory of Medicinal Chemical Biology, Haihe Laboratory of Cell Ecosystem, Nankai University, Tianjin, China; 2 Center for Cell Structure and Function, Shandong Provincial Key Laboratory of Animal Resistance Biology, College of Life Sciences, Shandong Normal University, Jinan, China; 3 Laboratory of Tissue Homeostasis, Haihe Laboratory of Cell Ecosystem, Tianjin, China

**Keywords:** Methylation, Cell division

## Abstract

The corneal epithelium is a constantly self-renewing, stratified squamous tissue that protects the inner eye from external stimuli. The organization of the corneal epithelium involves multiple biological activities, including basal cell proliferation and centripetal migration. However, the underlying molecular mechanisms remain unclear. Herein, we identify protein arginine methyltransferase 1 (PRMT1) as a key regulator of corneal epithelial homeostasis. We exploited an inducible *Prmt1* knockout mouse model and observed apparent disruption in the corneal epithelial homeostasis. PRMT1-deficient mice exhibited significant corneal epithelial thinning, as evidenced by histological and immunofluorescence staining with epithelium-specific markers. Further investigation showed that the epithelial thinning in these mice resulted from the dysfunction of basal cells. Immunostaining and 5-ethynyl-2’-deoxyuridine incorporation assays demonstrated that PRMT1 depletion significantly inhibited the proliferation and migration of basal cells, whereas no apparent apoptosis-related abnormalities were observed in these cells. Moreover, scratch wound healing assays revealed that knockdown of PRMT1 expression or inhibition of its catalytic activity significantly impaired the migration of corneal epithelial cells. Overall, our findings uncover a critical role for PRMT1 in controlling basal cell proliferation and migration to maintain corneal epithelial homeostasis, thereby providing potential therapeutic targets for the treatment of corneal diseases.

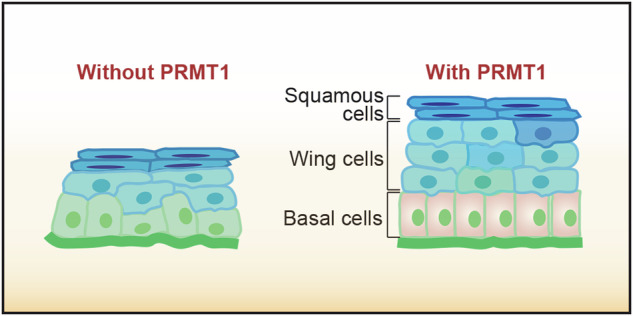

## Introduction

The cornea, located at the forefront of the eye, is a transparent structure crucial for maintaining the ocular barrier and refractive system [1, 2]. In mammals, the cornea comprises three cellular layers: the corneal epithelium, corneal stroma, and corneal endothelium, which are separated by Bowman’s membrane and Descemet’s membrane [3]. The corneal epithelium is composed of 6–8 layers of stratified epithelial cells, including squamous cells, wing cells, and basal cells. Among these, basal cells serve as the proliferative compartment, undergoing continuous proliferation, upward migration, and terminal differentiation to maintain corneal epithelial homeostasis [4–7]. This homeostatic process is vital for maintaining the transparency, smoothness, and protective barrier of the cornea, which are essential for proper light refraction and clear vision [8, 9]. The precise regulation of basal cell proliferation and migration is essential for the continuous renewal of the corneal epithelium, which is particularly crucial given its constant exposure to environmental stressors. Disruption of this delicate balance can lead to a cascade of pathological events, compromising the barrier function of the corneal epithelium and making it susceptible to microbial infections, resulting in conditions such as keratitis [10–12]. For instance, in cases of bacterial keratitis caused by *Pseudomonas aeruginosa*, the breakdown of epithelial integrity allows bacterial invasion, leading to severe corneal inflammation and potential vision loss [13]. In severe cases, such disruptions can progress to corneal ulcers or even perforation, ultimately causing blindness [14–16]. Therefore, investigating the molecular mechanisms that control corneal epithelial homeostasis is essential for the development of effective therapeutic strategies.

Protein arginine methylation, mediated by protein arginine methyltransferases (PRMTs), has emerged as a common and vital post-translational modification in tissue homeostasis maintenance [17–19]. PRMT1 is the predominant type I PRMT and catalyzes the formation of monomethylarginine (MMA) and asymmetric dimethylarginine (ADMA) on its substrates [20]. It has been reported that PRMT1 is responsible for approximately 85% of asymmetric arginine dimethylation in mammalian cells [21]. PRMT1-mediated methylation of these substrates regulates a diverse array of cellular processes, including transcriptional regulation, mRNA splicing, DNA damage and repair, metabolism remodeling, and signal transduction [21, 22]. Recent studies have highlighted an essential role for PRMT1 in the homeostasis of the pancreas and the hematopoietic system [23, 24]. However, it remains to be elucidated whether PRMT1 is involved in the modulation of corneal epithelial homeostasis.

In this study, bioinformatic analysis suggests a potential involvement of PRMT1 in corneal epithelial homeostasis. To investigate this possibility, we generated an inducible *Prmt1* knockout mouse model. Our results demonstrate that PRMT1 is essential for maintaining corneal epithelial homeostasis. Specifically, genetic ablation of *Prmt1* leads to significant thinning of both central and peripheral corneal epithelia. Further investigations reveal that this epithelial thinning induced by PRMT1 depletion is attributable to impaired activities of corneal epithelial basal cells, including reduced proliferation and compromised migration. Collectively, our findings establish that PRMT1 maintains corneal epithelial homeostasis by stimulating the proliferation and migration of corneal epithelial basal cells. These findings reveal a novel mechanism underlying corneal homeostasis and highlight PRMT1 as a potential therapeutic target for the treatment of corneal diseases.

## Results

### PRMT1 exhibits enriched expression in corneal epithelial basal cells

**Fig. 1:**
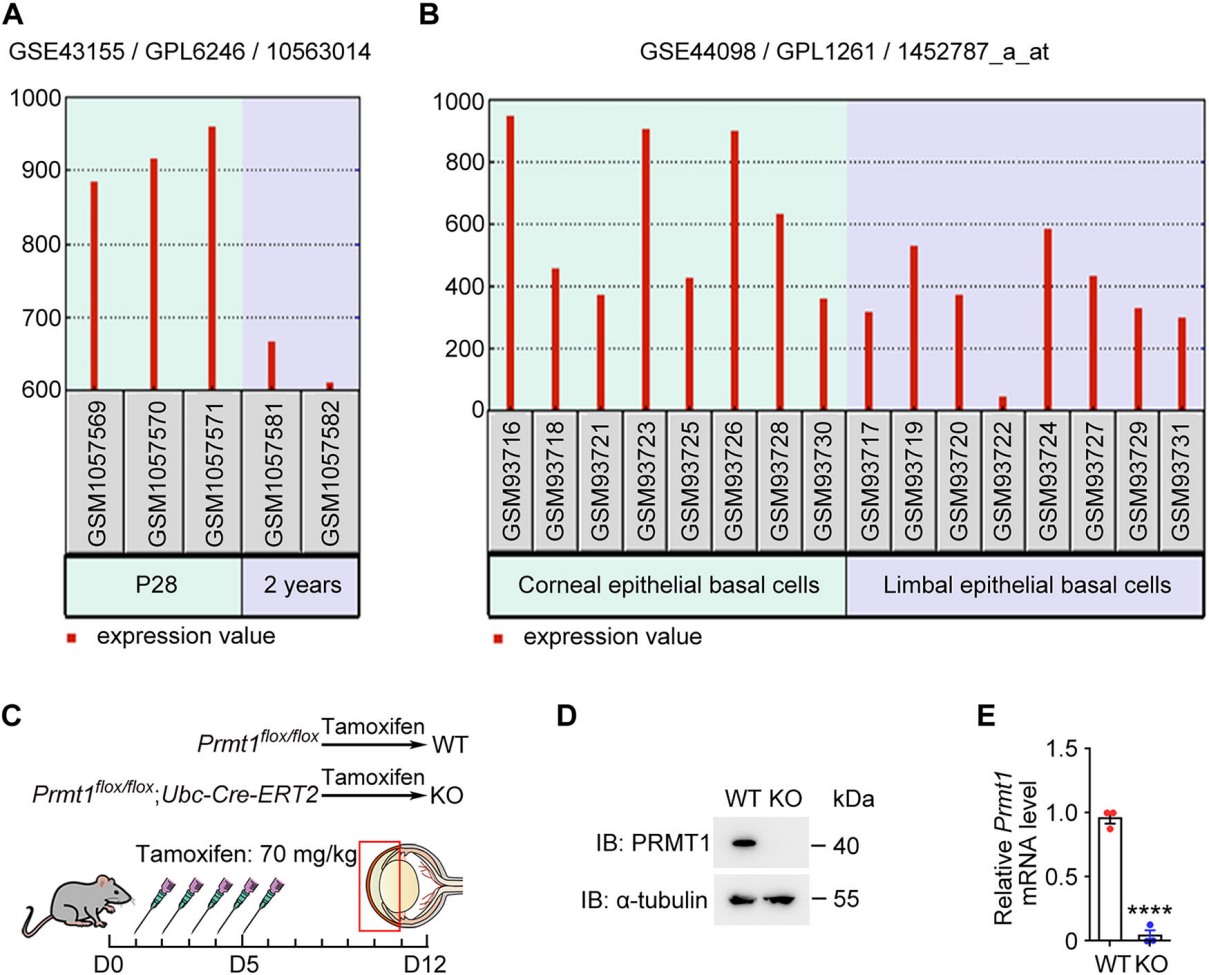
Elevated PRMT1 expression in corneal epithelial basal cells. **A** *Prmt1* mRNA expression in the whole corneas of juvenile (postnatal day 28) and aged (2-year-old) mice. Platform number GPL6246, GSE number GSE43155, *Prmt1* ID10563014. **B** *Prmt1* mRNA expression in the corneal epithelial basal cells and limbal basal cells. Platform number GPL1261, GSE number GSE4098, *Prmt1* ID 1452787_a_at. **C** Schematic diagram for the generation of *Prmt1* knockout (KO) mice. WT, wild-type. **D** Immunoblot analysis of PRMT1 and α-tubulin in the cornea of wild-type and *Prmt1* knockout mice. **E** RT-qPCR analysis showing *Prmt1* mRNA expression in the cornea of wild-type and *Prmt1* knockout mice (*n* = 3 independent experiments). Data are presented as mean ± SEM. ****p < 0.0001.

To investigate whether PRMT1 is involved in the regulation of corneal epithelial homeostasis, we analyzed the publicly available transcriptome datasets. In an expression profile of mouse corneal development and aging, we found that the *Prmt1* mRNA level in the cornea was higher in juvenile mice (postnatal day 28, P28) compared to that in aged mice (2-year-old) (Platform number GPL6246, GSE number GSE43155, *Prmt1* ID 10563014) [25] (Fig. 1A). Given the established age-related decline in corneal regenerative capacity [26, 27], this profiling dataset indicates the potential involvement of PRMT1 in the maintenance of corneal homeostasis. Moreover, in another dataset about the gene expression difference between limbal epithelial basal cells and corneal epithelial basal cells, we found that *Prmt1* mRNA expression was higher in corneal epithelial basal cells (Platform number GPL1261, GSE number GSE4098, *Prmt1* ID 1452787_a_at) [28] (Fig. 1B). This distinct expression pattern implicates PRMT1 in regulating corneal epithelial basal cell function. To explore this possibility, we exploited an inducible *Prmt1* knockout mouse model (*Prmt1*^*flox/flox*^*; Ubc*-*Cre-ERT2*). Since systemic *Prmt1* knockout leads to embryonic lethality [29], we collected corneas 7 days after 5 consecutive days of intraperitoneal tamoxifen injection to minimize the confounding effects of systemic perturbations (Fig. 1C). Immunoblotting and RT-qPCR confirmed the absence of PRMT1 at both the protein and mRNA levels in the cornea of *Prmt1* knockout mice (Fig. 1D, E).

### PRMT1 maintains the corneal epithelial integrity

**Fig. 2:**
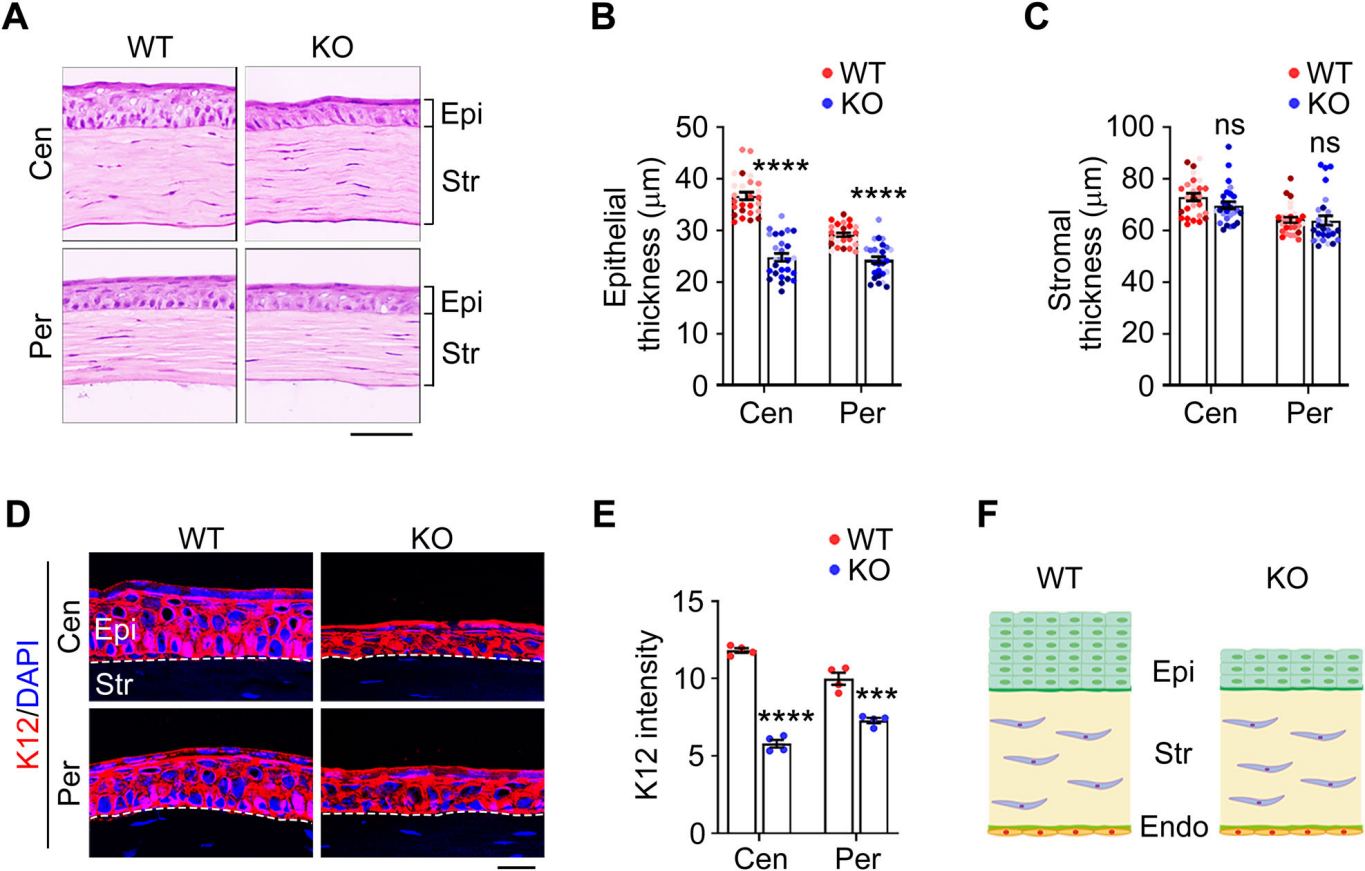
*Prmt1* knockout leads to mouse corneal epithelial thinning. H&E staining (**A**) and quantification of the central (Cen) and peripheral (Per) corneal epithelial thickness (**B**) and stromal thickness (**C**) of wild-type and *Prmt1* knockout mice (*n* = 27 fields from 6 mice). Epi, epithelium. Str, stroma. Scale bar, 50 μm. Immunofluorescence microscopy (**D**) and quantification of the K12 intensity (**E**) in the central and peripheral corneal epithelium of wild-type and *Prmt1* knockout mice (*n* = 4 mice). Scale bar, 20 μm. **F** Schematic illustration of the corneal epithelium (Epi), stroma (Str), and endothelium (Endo) in wild-type and *Prmt1* knockout mice. Data are presented as mean ± SEM. ***p < 0.001, ****p < 0.0001; ns, not significant.

To investigate whether PRMT1 plays a role in regulating corneal structure, the whole corneal histology was examined by microscopic analysis of hematoxylin and eosin (H&E)-stained sections. We measured and compared the thickness of the peripheral and central cornea, respectively, due to their thickness disparity indicated by the distribution profile [30]. *Prmt1* knockout mice exhibited a marked reduction in epithelial thickness, with decreases of approximately 35% in the central cornea and 20% in the peripheral cornea compared to wild-type controls (Fig. 2A, B). In contrast, this epithelial thinning occurred without any concomitant changes in stromal thickness, as demonstrated by precise measurements showing less than 5% variation between knockout and control groups (Fig. 2A, C). The endothelial monolayer maintained its characteristic hexagonal cellular morphology and showed no signs of structural compromise (Fig. 2A). To further confirm these findings, we conducted immunostaining for keratin 12 (K12), a well-established epithelial marker. Consistent with the H&E results, K12 staining revealed a pronounced decrease in epithelial thickness in both central and peripheral regions of *Prmt1* knockout corneas (Fig. 2D, E). Taken together, these data demonstrate that PRMT1 deficiency selectively disrupts corneal epithelial integrity without affecting the stromal or endothelial layers (Fig. 2F).

### PRMT1 maintains corneal epithelial stratification through modulation of the wing cell population

**Fig. 3:**
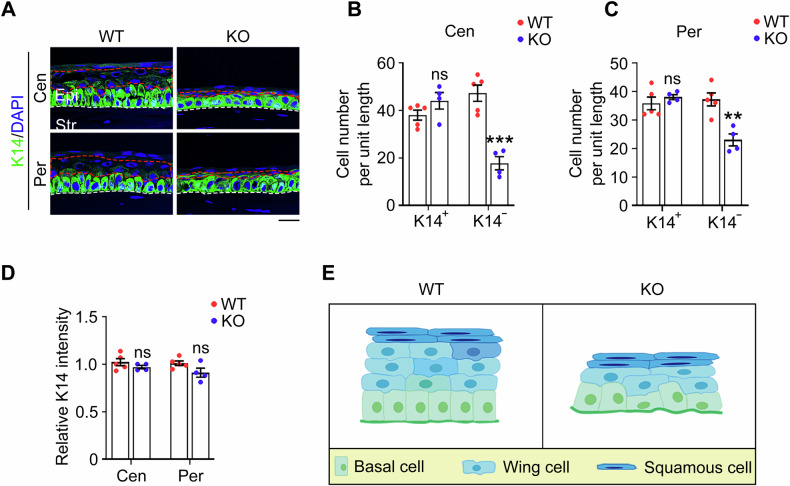
*Prmt1* knockout disrupts corneal epithelial homeostasis. **A** Immunofluorescence microscopy of the K14 staining in the central and peripheral corneal epithelium of wild-type (*n* = 5 mice) and *Prmt1* knockout mice (*n* = 4 mice). Scale bar, 20 μm. Quantification of K14-positive and K14-negative cells in the central (**B**) or peripheral (**C**) corneal epithelium of wild-type (*n* = 5 mice) and *Prmt1* knockout mice (*n* = 4 mice). **D** Quantification of the relative K14 intensity in the central and peripheral corneal epithelium of wild-type (*n* = 5 mice) and *Prmt1* knockout mice (*n* = 4 mice). **E** Schematic illustration of the corneal epithelium in wild-type and *Prmt1* knockout mice. Data are presented as mean ± SEM. **p < 0.01, ***p < 0.001; ns not significant.

The corneal epithelium is organized into 6–8 distinct cell layers, consisting of the superficial squamous cells, wing cells, and basal cells [31]. Among them, basal cells are the only cell type that is capable of proliferation, while wing cells and squamous cells are considered as terminally differentiated cells [32]. To determine the specific changes in the corneal epithelium of *Prmt1* knockout mice, we stained the cornea with an anti-K14 antibody, which specifically marks the basal cell layer. Intriguingly, immunofluorescence microscopy revealed no significant differences in K14 intensity or distribution in the basal cells between *Prmt1* knockout and wild-type mice (Fig. 3A–D). However, we observed a significant reduction in the number of terminally differentiated cells in the corneal epithelium of *Prmt1* knockout mice (Fig. 3B–D), indicated as K14-negative cells. Specifically, the reduction rate was approximately 60% in the central cornea and 40% in the peripheral cornea, compared to wild-type controls (Fig. 3B, C). Further data stratification demonstrated that this reduction was primarily attributed to a significant decrease in wing cell numbers, whereas squamous cell populations remained largely unaffected (Fig. 3A). Collectively, these results reveal an essential role of PRMT1 in maintaining corneal epithelial stratification, predominantly through the modulation of the wing cell population (Fig. 3E).

### PRMT1 is important for the proliferation and differentiation of corneal epithelial basal cells

**Fig. 4:**
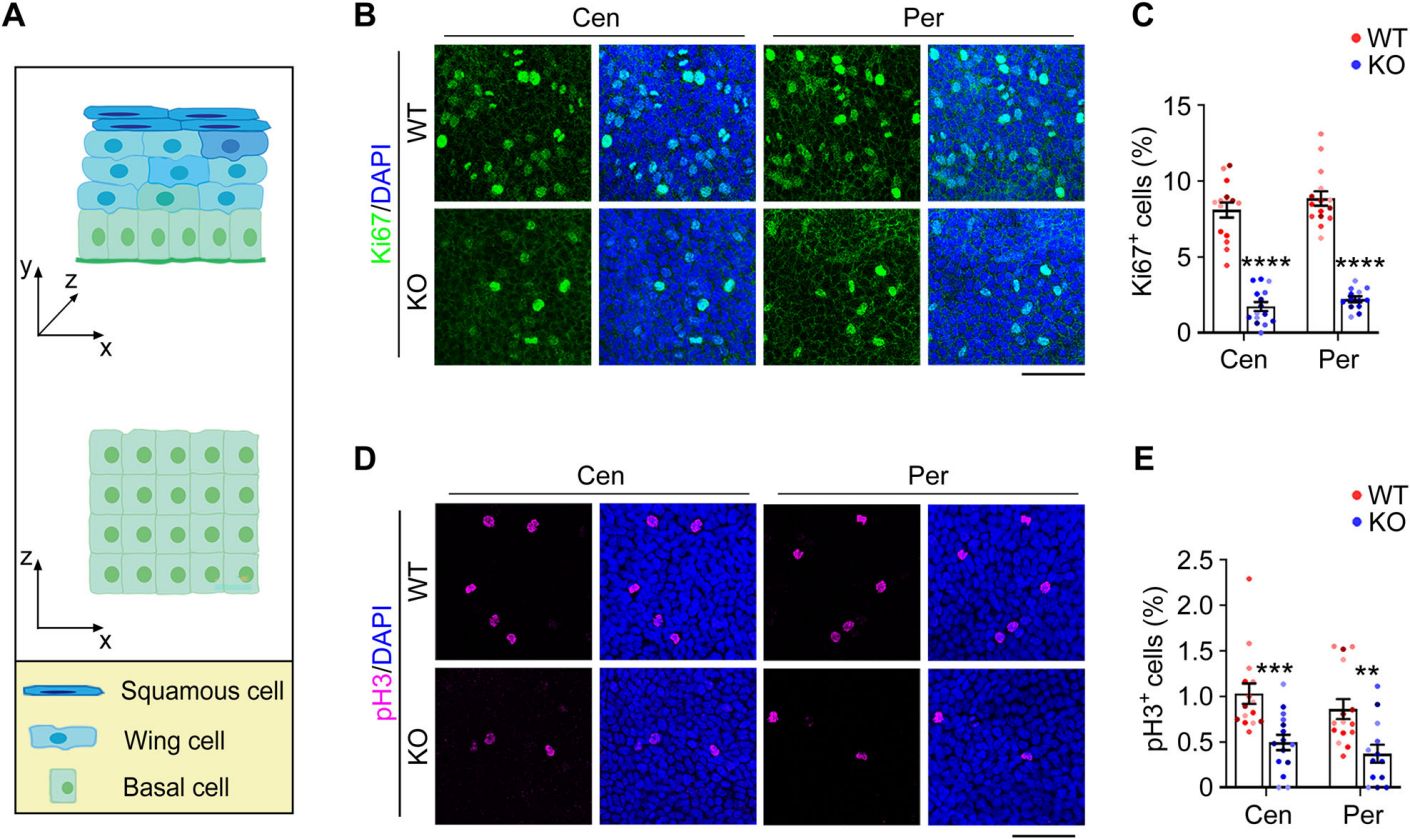
PRMT1 depletion inhibits corneal epithelial basal cell proliferation. **A** Schematic diagram of the stratified corneal epithelium (x–y plane) and the corneal epithelial basal cell layer (x–z plane). Immunofluorescence microscopy (**B**) and quantification of Ki67-positive cells (**C**, *n* = 13 fields from 5 mice) in the corneal epithelial basal cell layer of wild-type and *Prmt1* knockout mice. Scale bar, 50 μm. Immunofluorescence microscopy (**D**) and quantification of pH3-positive cells (**E**, *n* = 13 fields from 5 mice) in the corneal epithelial basal cell layer of wild-type and *Prmt1* knockout mice. Scale bar, 50 μm. Data are presented as mean ± SEM. **p < 0.01, ***p < 0.001, ****p < 0.0001; ns, not significant.

Given that terminally differentiated cells originate from proliferative basal cells [33], the observed corneal epithelial thinning and reduced wing cell population suggest potential dysfunction of basal cells in *Prmt1* knockout mice. To test this hypothesis, we first assessed epithelial basal cell proliferation by immunofluorescence staining of the whole cornea. We chose two distinct proliferation markers, Ki67 and phosphorylated histone 3 (pH3). Ki67 marks nuclei in all active phases of the cell cycle [34], while pH3 visualizes only the four actual phases of mitosis and late G2 [35]. The basal layer (Fig. 4A, x–z plane) was scanned, and the proportion of proliferating cells among the total basal cells was calculated. Immunofluorescence analysis of whole-mounted corneas revealed that PRMT1 depletion significantly reduced the proportion of Ki67-positive cells, with decreases of almost 80% in both central and peripheral corneas compared to wild-type controls (Fig. 4B, C). Consistent with these findings, pH3 staining demonstrated a comparable decrease in mitotic cells across the corneal epithelium, and the corresponding reduction rate was about 50% (Fig. 4D, E). Collectively, these results from independent proliferation markers establish that PRMT1 is essential for maintaining the proliferative capacity of corneal epithelial basal cells.

To further investigate whether PRMT1 depletion affects the differentiation of basal cells, we performed immunofluorescence staining for desmocollin 2 (DSC2), a marker specific for suprabasal layers of the corneal epithelium that reflects basal cell differentiation capacity [36]. In wild-type mice, DSC2 expression was robust in the suprabasal layers, suggesting normal differentiation of basal cells into wing cells (Fig. S1A, B). In contrast, PRMT1-depleted corneas exhibited a significant reduction in DSC2 intensity, indicating impaired differentiation of basal cells into suprabasal layers (Fig. S1B). Quantification of DSC2 intensity revealed a reduction of approximately 60% in both central and peripheral regions of the corneal epithelium (Fig. S1B). These findings demonstrate that PRMT1 depletion not only reduces the proliferative capacity of basal cells but also disrupts their differentiation into wing cells, thereby contributing to the observed thinning of the corneal epithelium.

### PRMT1 is essential for the migration of corneal epithelial basal cells

The corneal epithelial stratification requires coordinated basal cell proliferation and subsequent upward migration to form functional suprabasal wing cell layers [37]. The observed reduction in wing cell populations in *Prmt1* knockout mice prompted us to investigate potential migratory defects in epithelial basal cells.

**Fig. 5:**
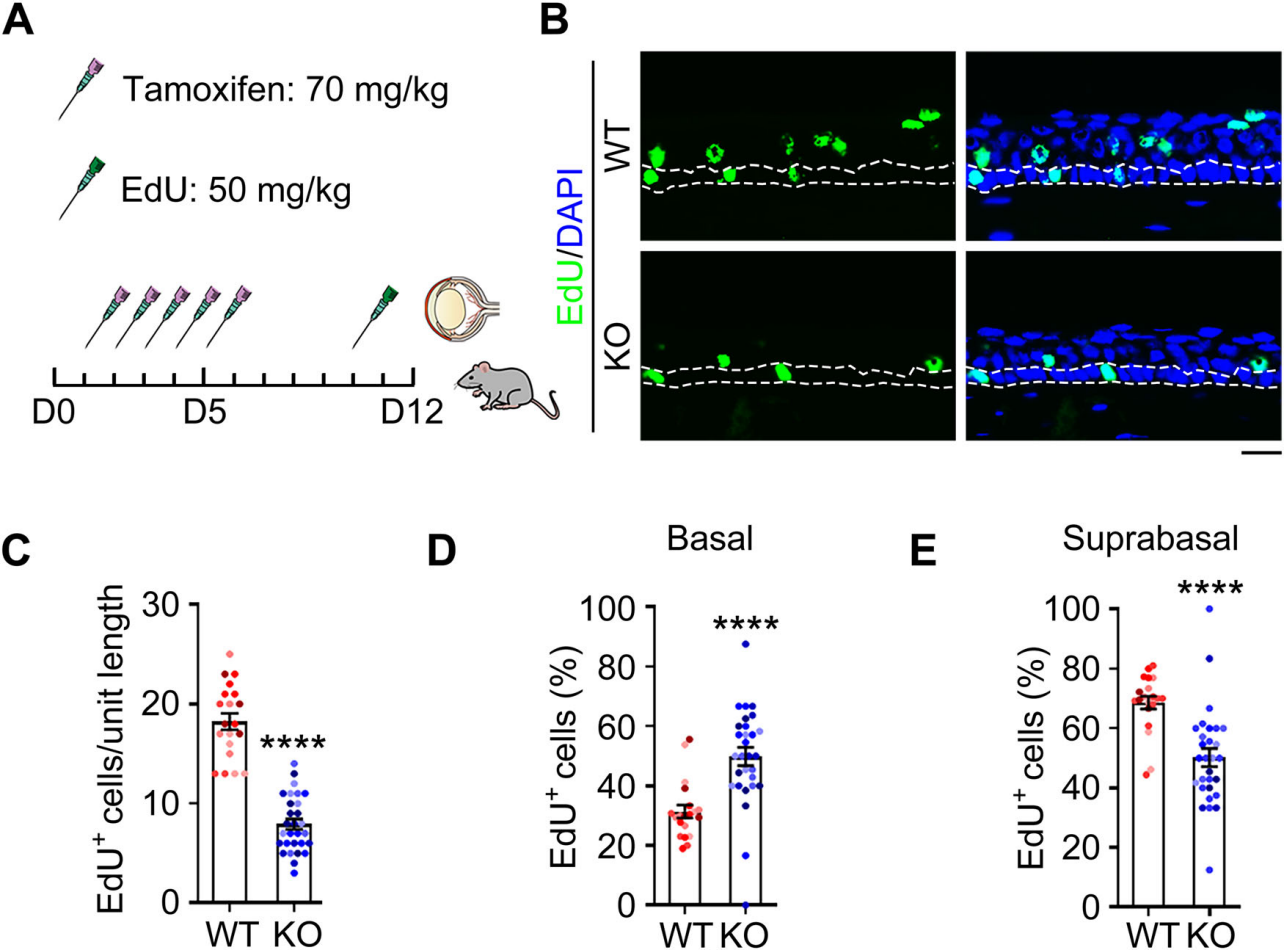
PRMT1 is essential for the migration of corneal epithelial basal cells in vivo. **A** Schematic illustration of EdU injection using *Prmt1* knockout mice. Immunofluorescence microscopy (**B**) and quantification of EdU-positive cells (**C**, *n* = 20 fields from 5 mice) in the corneal epithelium of wild-type and *Prmt1* knockout mice. Scale bar, 20 μm. Percentage of EdU-positive cells in the basal layer (**D**) and suprabasal layer (**E**) relative to the total EdU-positive cells in the corneal epithelium of wild-type and *Prmt1* knockout mice (*n* = 20 fields from 5 mice). Data are presented as mean ± SEM. ****p < 0.0001; ns not significant.

To examine the migration of epithelial basal cells, after 5 consecutive days of tamoxifen injection, mice were maintained for an additional 5 days and then subjected to an intraperitoneal injection of 50 mg/kg EdU to label proliferating cells (Fig. 5A). Since basal cell is the only cell type with proliferation capability in the corneal epithelium [38, 39], EdU-positive cells in the upper layers (suprabasal cells) represent cells that have migrated upward from the basal layer (Fig. 4A, x–y plane). We found that the number of EdU-positive cells in the entire corneal epithelium of *Prmt1* knockout mice was significantly lower than that in wild-type mice (Fig. 5B, C), confirming that PRMT1 is critical for the proliferation of corneal epithelial basal cells. To quantify the migration capacity of basal cells, we calculated the proportion of EdU-positive basal and suprabasal cells, respectively, among the total EdU-positive cells. We found that PRMT1 depletion increased the proportion of EdU-positive basal cells among total EdU-positive cells; however, the proportion of EdU-positive suprabasal cells among total EdU-positive cells was significantly decreased upon PRMT1 depletion (Fig. 5D, E). These results suggest a significant inhibition of basal cell migration in *Prmt1* knockout mice.

### PRMT1 regulates corneal epithelial cell migration through its methyltransferase activity

Building upon the above in vivo findings demonstrating the essential role of PRMT1 in corneal epithelial basal cell migration, we next sought to determine whether this function is cell-intrinsic and dependent on its enzymatic activity. Using human corneal epithelial (HCE2) cells, we employed complementary genetic and pharmacological approaches to dissect the role of PRMT1 in cell migration.

**Fig. 6:**
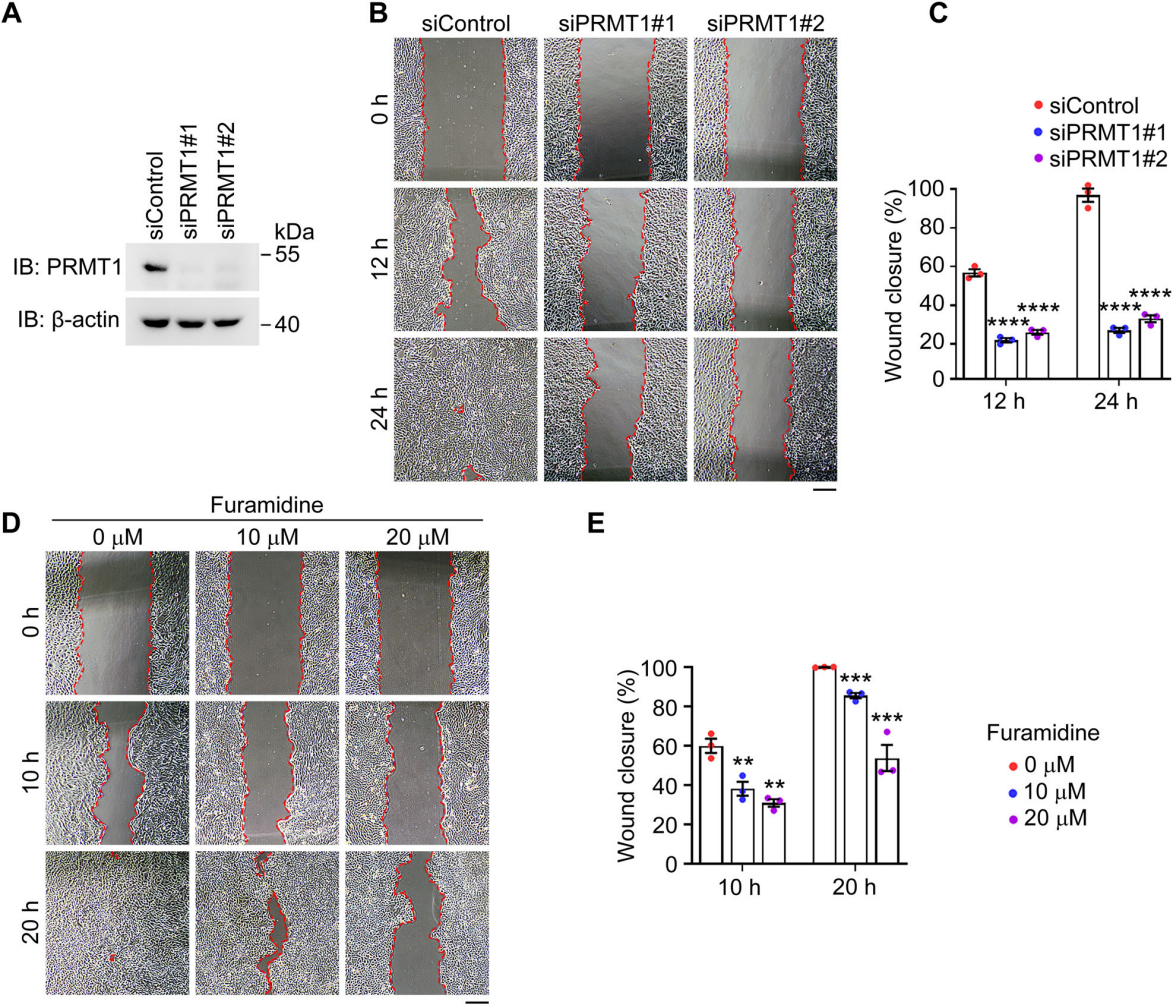
PRMT1 is pivotal for the migration of corneal epithelial cell in vitro. **A** Immunoblot analysis of PRMT1 and β-actin in HCE2 cells transfected with control or PRMT1 siRNAs. Phase-contrast images (**B**) and quantification of the percentage of wound closure (**C**, *n* = 3 independent experiments) in scratch wound healing assays, using HCE2 cells transfected with control or PRMT1 siRNAs. Scale bars, 200 μm. Phase-contrast images (**D**) and quantification of the percentage of wound closure (**E**, *n* = 3 independent experiments) in scratch wound healing assays, using HCE2 cells treated with different concentrations of furamidine. Scale bars, 200 μm. Data are presented as mean ± SEM. **p < 0.01, ***p < 0.001, ****p < 0.0001; ns, not significant.

We performed siRNA-mediated knockdown of PRMT1 in HCE2 cells and assessed the migration ability using scratch wound assays. Corresponding uncropped western blots presenting knockdown efficiency are included in the supplementary files. Briefly, after cells reached about 90% confluency, wounds were introduced by scratching, and the percentage of wound closure was quantified after a certain time. Remarkably, PRMT1 depletion significantly impaired wound closure (Fig. 6A–C), recapitulating the migratory defect observed in PRMT1-deficient corneal epithelium in vivo. This finding strongly suggests that PRMT1 regulates epithelial cell migration through intrinsic cellular mechanisms. To determine whether this regulatory function depends on the methyltransferase activity of PRMT1, we employed pharmacological inhibition using furamidine, a selective PRMT1 inhibitor [40]. Consistent with the genetic approach, furamidine treatment substantially reduced the migration of HCE2 cells (Fig. 6D, E). These results demonstrate that PRMT1-dependent methylation events are essential for proper epithelial cell migration.

### PRMT1 promotes the monomethylation of microtubule-associated protein 4 (MAP4) in corneal epithelial cells

**Fig. 7:**
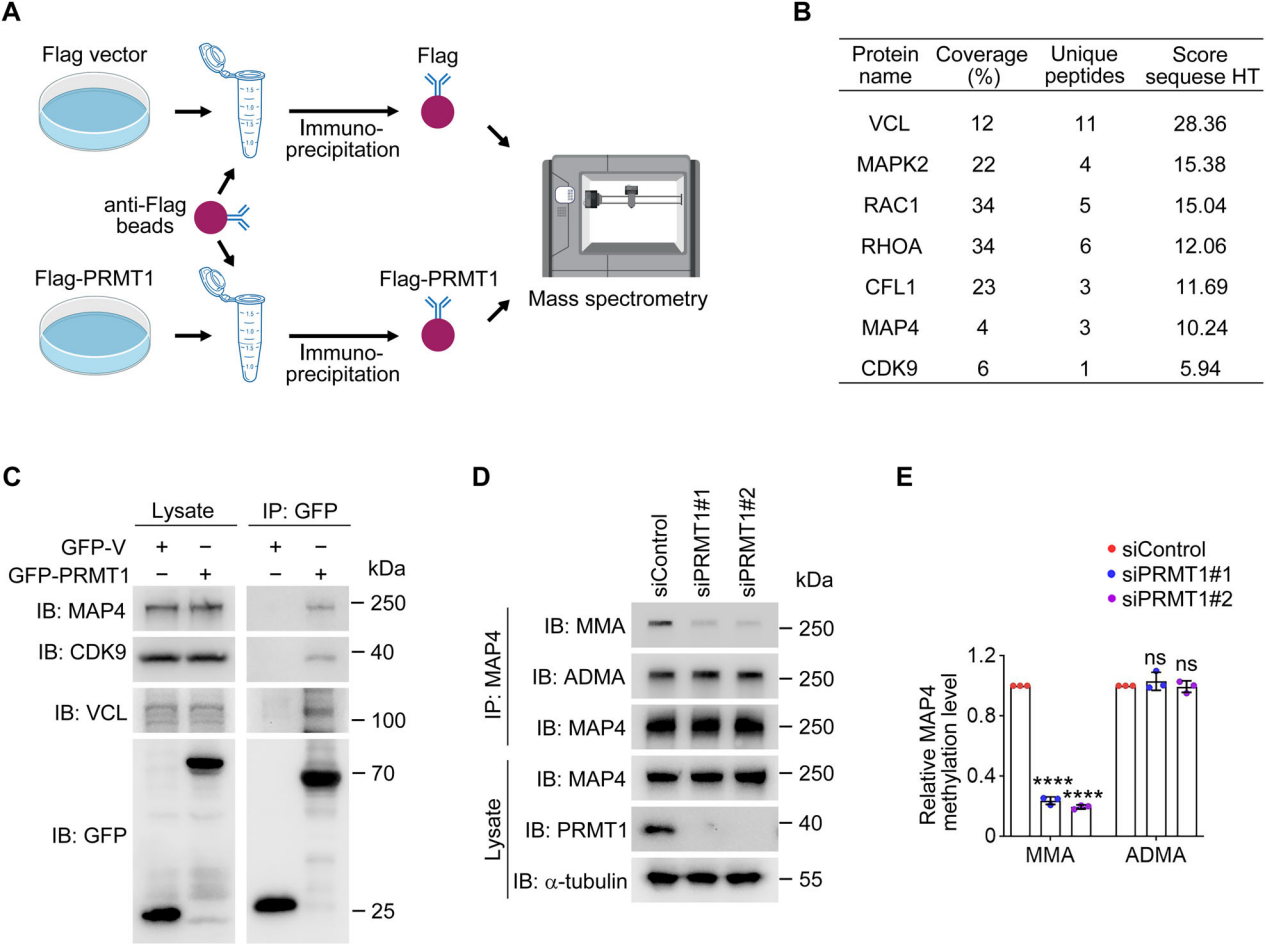
PRMT1 mediates the monomethylation of MAP4. **A** Schematic illustration of sample preparation for mass spectrometry. **B** Potential substrates of PRMT1 identified by mass spectrometry. **C** Immunoprecipitation and immunoblotting showing the interaction of GFP-PRMT1 with endogenous MAP4, CDK9, and VCL. Immunoprecipitation and immunoblotting showing the monomethylation and asymmetric dimethylation of MAP4 in HCE2 cells transfected with control or PRMT1 siRNAs (**D**). The relative MAP4 methylation level was determined by densitometry (**E**, *n* = 3 independent experiments). MMA monomethylarginine, ADMA asymmetric dimethylarginine. Data are presented as mean ± SEM. ****p < 0.0001; ns not significant.

To explore the molecular mechanisms by which PRMT1 regulates corneal epithelial cell proliferation and migration, we immunoprecipitated Flag-PRMT1 from total cell lysates of HEK293T cells (Fig. 7A). Mass spectrometry revealed several proteins known to regulate cell proliferation and migration, each validated by multiple unique peptides (Table S1). To prioritize candidates relevant to the corneal epithelium, the single-cell RNA sequencing dataset GSE155683, which characterizes the human cornea across developmental and adult stages, was obtained from the Gene Expression Omnibus (GEO) database [41]. This approach revealed seven proteins with both high interaction scores and elevated expression in the corneal epithelium: MAP4, cyclin-dependent kinase 9 (CDK9), vinculin (VCL), cofilin (CFL1), Ras homolog family member A (RHOA), receptor for activated C kinase 1 (RAC1), and mitogen-activated protein kinase 2 (MAPK2) (Fig. 7B).

Among these candidates, we focused on MAP4, CDK9, and VCL due to their significant interactions with PRMT1 (Figs. 7C and S2A). To determine which protein(s) is the downstream substrate of PRMT1, we assessed the levels of MMA and ADMA in HCE2 cells following PRMT1 knockdown. Strikingly, PRMT1 depletion led to a significant reduction in the MMA level of MAP4, while its ADMA level remained unchanged (Fig. 7D, E). In contrast, the methylation levels of CDK9 and VCL were not significantly altered by PRTM1 depletion (Fig. S2B, C). These findings suggest that MAP4 is a substrate of PRMT1 in the regulation of corneal epithelial homeostasis, likely through its monomethylation.

## Discussion

The corneal epithelium, located at the outermost layer of the cornea, plays a critical role in maintaining structural and functional integrity essential for normal vision [42, 43]. Corneal epithelial basal cells originate from limbal stem cells [6], and are crucial for the maintenance of corneal epithelial homeostasis. However, the underlying molecular mechanisms remain poorly understood. In this study, we have identified PRMT1 as a key regulator of corneal epithelial homeostasis and found that depletion of PRMT1 leads to a significantly thinner corneal epithelium. The decrease in corneal epithelial thickness is known to compromise the corneal barrier function, leading to corneal infection and inflammation [44]. Indeed, we have observed corneal epithelial ulcers and even corneal perforations in the later stages of *Prmt1* knockout mice (Fig. S3). Therefore, while PRMT1 depletion initially affects corneal epithelial homeostasis, persistent PRMT1 deficiency may also have detrimental effects on the corneal stroma and endothelium.

Corneal epithelium, as a constantly self-renewing stratified squamous tissue, protects the inner eye from external stimuli [45]. Appropriate regulation of cells in this exquisite structure guarantees its protective function and refractive properties [46]. Corneal epithelium homeostasis is a comprehensive process, and the precise and proper arrangement of corneal epithelium involves multiple biological activities, including cell proliferation, migration, differentiation, and apoptosis [4–7]. Basically, when corneal epithelial cells initiate the apoptosis process in response to damage or desquamation, the neighboring cells would release growth factors to enhance proliferation and migration [47]. In the present study, we demonstrated that PRMT1 enhances the proliferation and differentiation of basal cells, and promotes corneal epithelial cell migration. However, when we analyzed cell apoptosis by terminal deoxynucleotidyl transferase dUTP nick end labeling (TUNEL), we did not observe a significant difference in apoptosis rates between wild-type and *Prmt1* knockout mice in corneal epithelial basal cells (Fig. S4A, B). This selective involvement suggests PRMT1 participates in the active maintenance phase of epithelial turnover rather than cell death pathways.

As the main type I PRMT in mammals, PRMT1 has been implicated in the pathological processes of various human diseases [21]. In the present study, we have demonstrated that PRMT1 is essential for maintaining corneal epithelial homeostasis. Specifically, PRMT1 depletion results in a significantly thinner corneal epithelium. In clinical ophthalmology, corneal epithelial thickness is a critical biomarker for ocular health [48]. Alterations in the corneal epithelial thickness are closely associated with the development of various ocular surface disorders, such as dry eye, keratoconus, diabetic keratopathy, and persistent corneal epithelial defects [48–53]. Therefore, future studies should investigate whether PRMT1 is involved in the pathogenesis of corneal diseases by regulating corneal epithelial thickness. Last but not least, recent studies have shown that multiple signaling pathways, including PI3K/AKT, EGFR, Wnt/β-catenin, and Hippo-Yap, are involved in the corneal epithelial homeostasis [54–57]. Future studies should focus on identifying specific PRMT1 substrates and their methylation-dependent interactions within these pathways.

## Materials and methods

### Generation of knockout mice

*Prmt1*^flox/flox^ mice were kindly provided by Dr. Shilai Bao (Institute of Genetics and Developmental Biology, Chinese Academy of Sciences). *Ubc-Cre-ERT2* mice were purchased from the Jackson Laboratory. All mice were maintained in the C57BL/6 J background. To generate *Prmt1* knockout mice, tamoxifen (Sigma, T5648) was dissolved in corn oil at a concentration of 20 mg/mL and injected into the abdominal cavity of 8-week-old male mice (*Prmt1*^flox/flox^; *Ubc-Cre-ERT2*) at a dose of 70 mg/kg body weight for 5 consecutive days. Tissues were harvested at 7 days after the final injection. *Prmt1*^flox/flox^ mice receiving the same tamoxifen treatment served as the wild-type group for the study. All mice were bred in the specific pathogen free barrier of the Experimental Animal Center at Nankai University. Animal experiments were conducted with randomization and blinding.

### Cell culture and transfection

HCE2 cells were purchased from American Type Culture Collection (ATCC, Manassas, VA, USA). HCE2 cells were cultured in Dulbecco’s Modified Eagle’s Medium (DMEM). All cultures were supplemented with 10% fetal bovine serum (C04001-500, VivaCell Biosciences) and incubated at 37 °C in a humidified atmosphere containing 5% CO_2_. siRNAs were transfected using Lipofectamine RNAiMAX (13778030, Invitrogen), according to the manufacturer’s instructions.

### Antibodies

Antibodies for immunofluorescence staining include anti-K12 (ab185627, Abcam), anti-K14 (10143-1-AP, Proteintech), anti-Ki67 (ab279653, Abcam), anti-pH3 (06-570, Millipore), anti-DCS2 (13876-1-AP, Proteintech), Alexa Fluor™ 568 donkey anti-mouse IgG (H + L) (A10037, Invitrogen), Alexa Fluor™ 488 donkey anti-mouse IgG (H + L) (A21202, Invitrogen), Alexa Fluor™ 568 donkey anti-rabbit IgG (H + L) (A10042, Invitrogen), Alexa Fluor™ 488 donkey anti-rabbit IgG (H + L) (A21206, Invitrogen), and Alexa Fluor™ 647 donkey anti-mouse IgG (H + L) (A31571, Invitrogen). Antibodies for immunoblotting include anti-PRMT1 (2449S, Cell Signaling Technology), anti-β-actin (AC026, ABclonal), anti-GFP (ab0005, Abways), anti-MAP4(11229-1-AP, Proteintech), anti-VCL (ab18058, Abcam), anti-CDK9 (11705-1-AP, Proteintech), anti-MMA (#8015, Cell Signaling Technology), anti-ADMA (#13522, Cell Signaling Technology), anti-CFL1(10960-1-AP, Proteintech), anti-MAPK2 (11257-1-AP, Proteintech), anti-RAC1 (24072-1-AP, Proteintech), and anti-RHOA (10749-1-AP, Proteintech).

### Immunofluorescence microscopy

For whole corneal staining, corneas were dissected along the limbus, permeabilized with 0.5% Triton X-100 for 3 min, and fixed in 4% paraformaldehyde for 20 min. Samples were blocked in 4% bovine serum albumin (BSA) with 0.5% Triton X-100 for 1 h, and then sequentially incubated with primary antibodies, secondary antibodies, and DAPI (D5942, Sigma). For corneal section staining, frozen tissue sections were briefly rinsed with phosphate-buffered saline (PBS) for 1 min, permeabilized with 0.1% Triton X-100 for 1 min, fixed with 4% paraformaldehyde for 15 min, and further permeabilized with 0.3% Triton X-100 for 15 min. Blocking was performed using 4% BSA containing 0.1% Triton X-100 for 1 h, followed by incubation with primary antibodies, secondary antibodies, and DAPI.

### Immunoprecipitation and immunoblotting

Cells were lysed using a lysis buffer containing Tris (20 mM, pH 7.5), NaCl (150 mM), EDTA (1 mM), EGTA (1 mM), Sodium pyrophosphate (1.25 mM), glycerin (10%), NP40 (1%), and complete EDTA-free protease cocktail (04693132001, Roche), followed by centrifugation at 12,000 rpm for 20 min at 4 °C. To perform immunoprecipitation, the lysate was incubated with 5 µL nanobody magarose beads at 4 °C for 4–6 h. After incubation, the beads were washed 8 times, and the proteins captured by the beads were subsequently analyzed through SDS-PAGE and immunoblotting. During the immunoblotting procedure, the proteins were first separated via SDS-PAGE and then transferred onto nitrocellulose membranes (66485, Pall Corporation). These membranes were blocked using Tris-buffered saline containing 0.2% Tween-20 and 5% skim milk. The blocked membranes were then incubated with primary antibodies diluted in the blocking solution for either 2 h at room temperature or overnight at 4 °C. Following four washes in Tris-buffered saline with Tween-20, the membranes were treated with secondary antibodies conjugated to horseradish peroxidase for 45 min at room temperature. The presence of bound antibodies was detected using an enhanced chemiluminescence detection reagent.

### RNA preparation and RT-qPCR

Corneal samples were used for the isolation of total RNAs following the manufacturer’s protocol using the TRIzol reagent (15596018, Thermo Fisher Scientific). The isolated RNA was then subjected to cDNA synthesis using M-MLV reverse transcriptase (M1701, Promega). For RT-qPCR analysis, triplicate reactions were performed using FastStart Universal SYBR Green Master (Rox) (04913914001, Roche), following standard protocol. The expression levels of target genes were normalized to β-actin to account for any sample-to-sample variation, using specific primers (PRMT1 forward: 5′-TACTACTTTGACTCCTATGCCCA-3′; PRMT1 reverse: 5′-ATGCCGATTGTGAAACATGGA-3′; β-actin forward: 5′-CAGAAGGAGATTACTGCTCTGGCT-3′; β-actin reverse: 5′-TACTCCTGCTTGCTGATCCACATC-3′).

### Mass spectrometry

To identify PRMT1-interacting proteins, whole-cell lysates were prepared from HEK293T cells overexpressing either the Flag vector or Flag-PRMT1. The lysates were immunoprecipitated by incubation with Flag antibody-conjugated agarose beads at 4 °C overnight. The immunoprecipitated proteins were then resolved by SDS-PAGE and analyzed by mass spectrometry using a Q Exactive HF mass spectrometer (Thermo Fisher Scientific). Mass spectrometric data were processed and analyzed using Proteome Discoverer 2.4 by PTM Biolabs (Hangzhou, China).

### Histological analysis

Eye samples were embedded in the OCT tissue freezing medium (4583, Sakura) and then sliced along the sagittal plane to prepare 6 μm sections for histological analysis as previously described [58]. To measure corneal thickness, sections with the largest diameter were selected. The prepared cryosections were stained with the H&E solution (G1120, Solarbio). Images were then captured and analyzed using a DM3000 microscope (Leica, Wetzlar).

### EdU incorporation assay

The proliferation of corneal epithelial basal cells was assessed using the BeyoClick EdU Cell Proliferation Kit with Alexa Fluor 488 (C0071S, Beyotime). Mice were intraperitoneally injected with EdU (50 mg/kg). After 48 h, eyeballs were harvested and embedded in the OCT tissue freezing medium. Cryosections were fixed and permeabilized as described above. Click chemistry was performed following the manufacturer’s instructions. Sections were washed with 4% BSA 3 times and incubated with DAPI before mounting.

### TUNEL staining

Cryosections were hydrated, prepermeabilized, fixed, and permeabilized as previously described [59]. TUNEL staining was performed using the DeadEnd Fluorometric TUNEL System Kit (G3250, Promega) according to the manufacturer’s instructions. Briefly, sections were incubated with 100 µL of the equilibration buffer at room temperature for 10 min. After removing the buffer, 50 µL of the rTdT incubation solution was added and incubated for 1 h at 37 °C in the dark. The reaction was terminated, and sections were washed 3 times with PBS, incubated with DAPI, and mounted.

### Scratch wound healing assay

Cells were seeded in 12-well plates, and the scratch wound healing assay was performed as previously described [60]. Briefly, cells were allowed to reach approximately 90% confluency. Wounds were then introduced by scratching the monolayer with a 200 µL pipette tip. After scratching, the cells were washed 3 times with PBS to remove cell debris. The wells were then replenished with medium containing 1% FBS and incubated for certain time. The percentage of wound closure was quantified using the ImageJ software based on photographs of the wounded areas taken with an inverted microscope.

### Statistical analysis

Statistical evaluations were performed using the ImageJ software and GraphPad Prism 9.0.0 (GraphPad Software). All experimental findings were presented as mean ± SEM. Each set of experiments was repeated at least 3 times. In cases where the data conformed to a normal distribution, Student’s *t*-test was employed to discern differences between two distinct groups, whereas an ordinary one-way analysis of variance (ANOVA) was utilized for comparisons involving three or more groups. Significance was ascribed to *p*-values < 0.05.

## Supplementary information

Supplementary information

uncropped western blots

Table S1

## Data Availability

Original data are available from the corresponding author upon reasonable request. The uncropped original western blots are shown in the supplementary files.
