# Supplementary material for: Protein arginine methyltransferase 1 stimulates basal cell proliferation and migration to maintain corneal epithelial homeostasis
Source: Cell Death Discov. 2025 Aug 15;11:385. doi: 10.1038/s41420-025-02684-6 (PMC12356924; doi:10.1038/s41420-025-02684-6)
Supplement: Supplementary file 1 — Supplementary information [file 41420_2025_2684_MOESM1_ESM.docx]

**Supplementary information**

**Protein arginine methyltransferase 1 stimulates basal cell proliferation and migration to maintain corneal epithelial homeostasis**

Jia Yang^1,4^, Mingzheng Hu^1,4^, Mulin Yang^1^, Hua Ni^1^, Jun Zhou^1,2^, Dengwen Li^1,^*, Jie Ran^2,^*, Min Liu^3,^*

^1^Department of Genetics and Cell Biology, College of Life Sciences, State Key Laboratory of Medicinal Chemical Biology, Haihe Laboratory of Cell Ecosystem, Nankai University, Tianjin 300071, China.

^2^Center for Cell Structure and Function, Shandong Provincial Key Laboratory of Animal Resistance Biology, College of Life Sciences, Shandong Normal University, Jinan 250014, China.

^3^Laboratory of Tissue Homeostasis, Haihe Laboratory of Cell Ecosystem, Tianjin 300462, China

^4^These authors contributed equally to this work.

*Correspondence: dwli@nankai.edu.cn (D.L.); jran@sdnu.edu.cn (J.R.); minliu@nankai.edu.cn (M.L.).


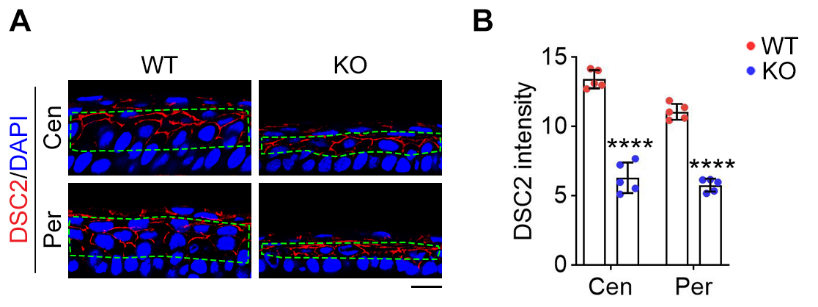


**Figure S1. PRMT1 depletion decreases the differentiation of corneal epithelial basal cells.**

**(A, B)** Immunofluorescence microscopy (A) and quantification of the DSC2 intensity (B) in the central and peripheral corneal epithelium of wild-type and *Prmt1* knockout mice (n = 5 mice). Scale bar, 20 μm.

Data are presented as mean ± SEM. ****p < 0.0001.


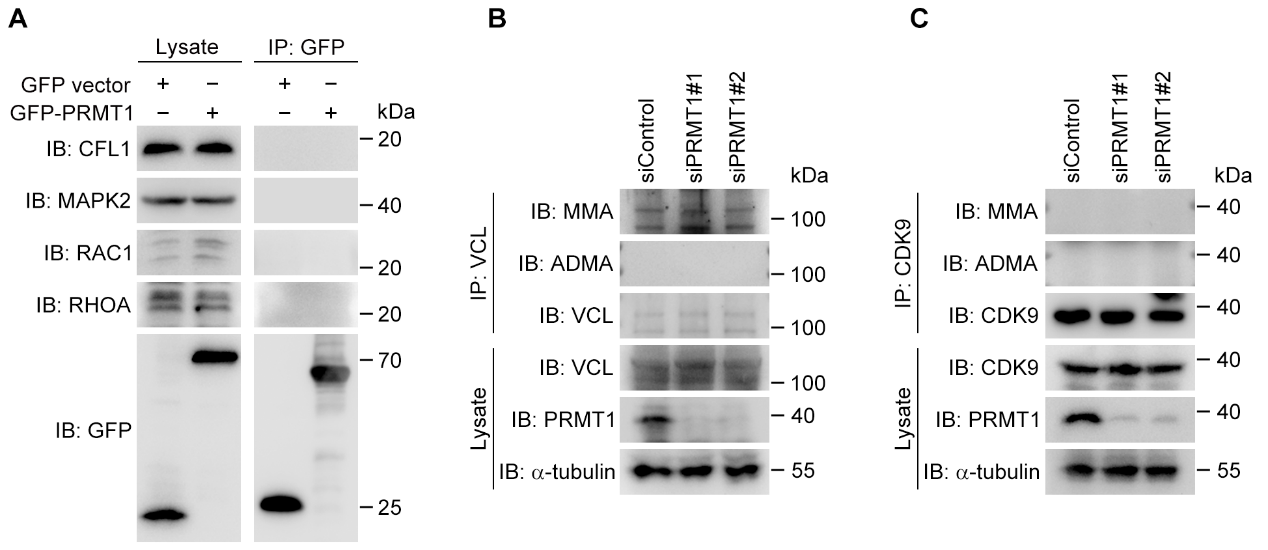


**Figure S2. Identification of PRMT1 substrates.**

**(A)** Immunoprecipitation and immunoblotting of the interaction between GFP-PRMT1 and CFL1, MAPK2, RAC1, and RHOA.

**(B, C)** Immunoprecipitation and immunoblotting of the monomethylation and asymmetric dimethylation of VCL (B) and CDK9 (C) in HEK293T cells transfected with control or PRMT1 siRNAs.


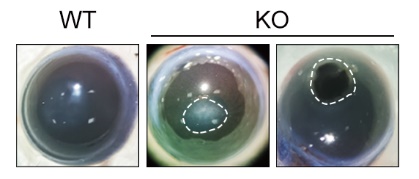


**Figure S3. Corneal epithelial ulcer and perforation in *Prmt1* knockout mice.**


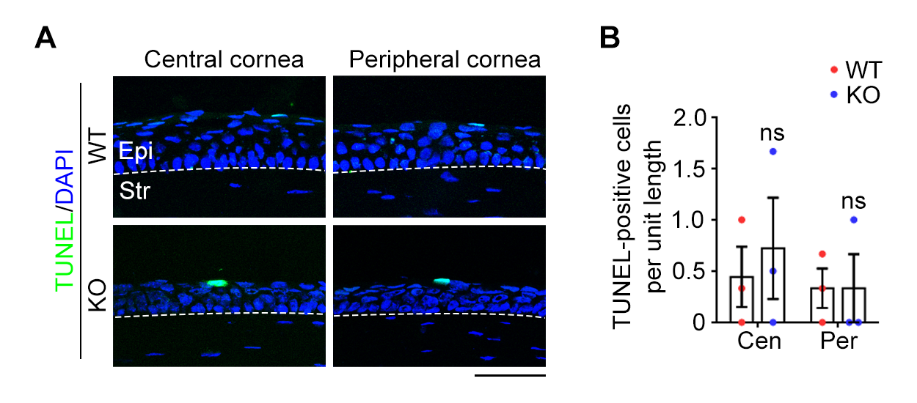


**Figure S4. Depletion of PRMT1 does not affect the percentage of apoptosis in corneal epithelial cells.**

**(A, B)** TUNEL staining (A) and quantification of TUNEL-positive cells (B, n = 3 independent experiments) for the corneal epithelium in wild-type and *Prmt1* knockout mice. Scale bar, 20 μm.

Data are presented as mean ± SEM. ns, not significant.
