# Supplementary figures and images for: Protein arginine methyltransferase 1 stimulates basal cell proliferation and migration to maintain corneal epithelial homeostasis
Source: Cell Death Discov. 2025 Aug 15;11:385. doi: 10.1038/s41420-025-02684-6 (PMC12356924; doi:10.1038/s41420-025-02684-6)

Figure 1D

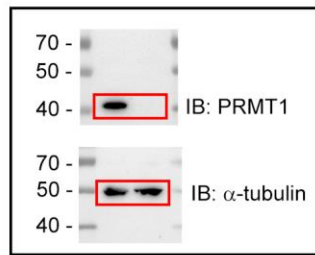

Figure 7C

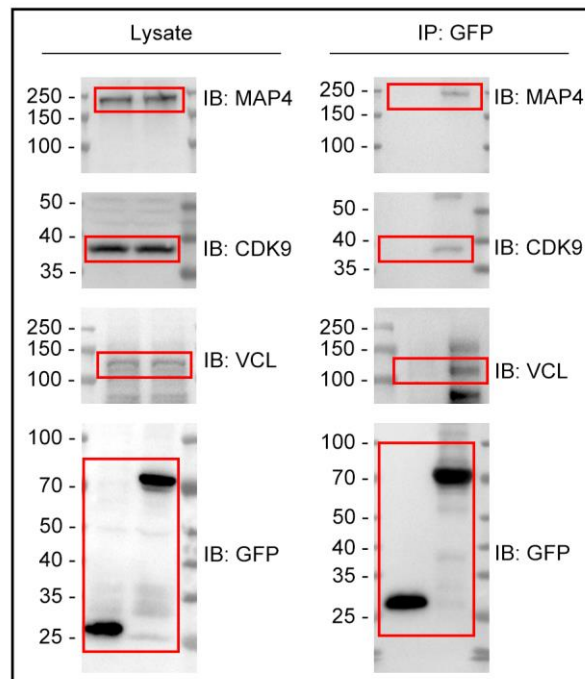

Figure 6A

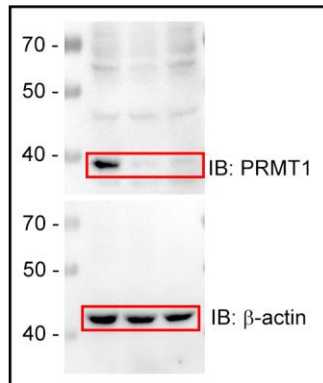

Figure 7D

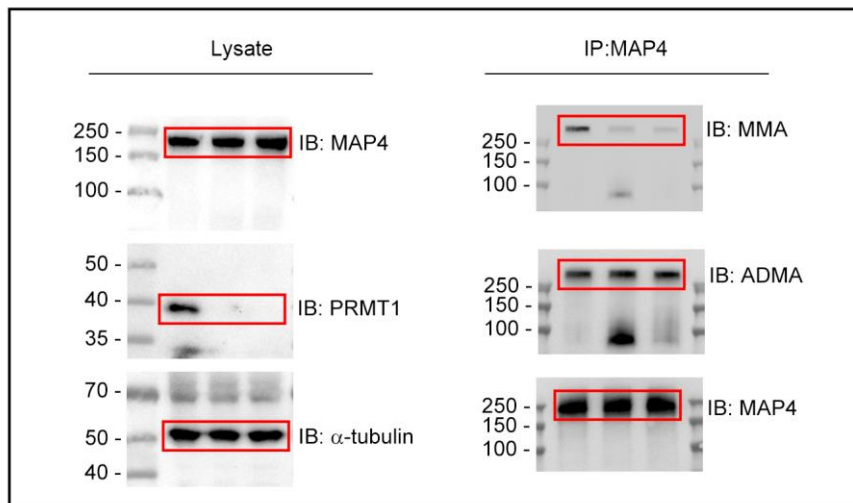

Figure S2A

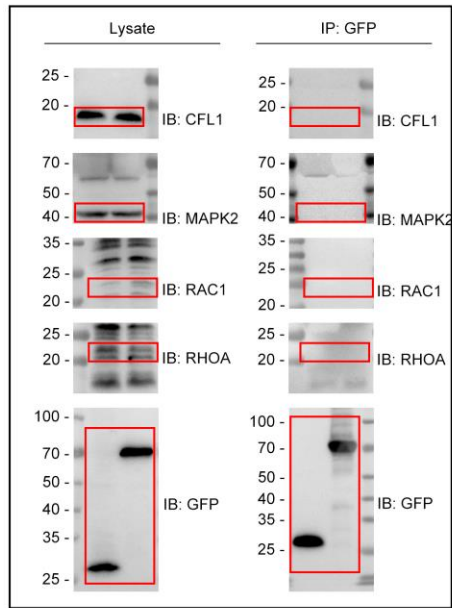

Figure S2B

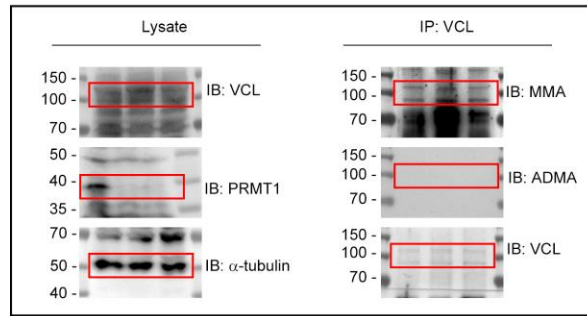

Figure S2C

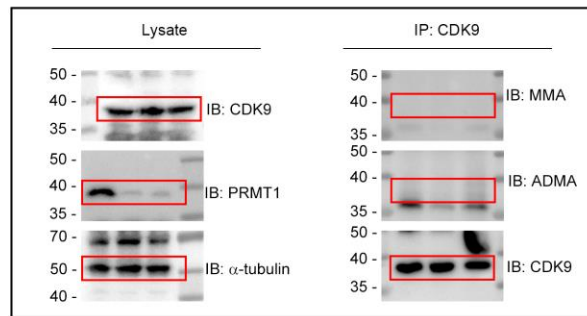

Supplement: Supplementary file 2 — uncropped western blots [file 41420_2025_2684_MOESM2_ESM.pdf]
